# Implementation of a Web-Based Tool for Shared Decision-making in Lung Cancer Screening: Mixed Methods Quality Improvement Evaluation

**DOI:** 10.2196/32399

**Published:** 2022-04-01

**Authors:** Julie Lowery, Angela Fagerlin, Angela R Larkin, Renda S Wiener, Sarah E Skurla, Tanner J Caverly

**Affiliations:** 1 Center for Clinical Management Research Ann Arbor VA Healthcare System Ann Arbor, MI United States; 2 Department of Population Health Sciences University of Utah School of Medicine Salt Lake City, UT United States; 3 Informatics Decision-Enhancement and Analytics Sciences Center for Innovation VA Salt Lake City Healthcare System Salt Lake City, MI United States; 4 Center for Healthcare Organization & Implementation Research VA Boston Healthcare System Boston, MA United States; 5 The Pulmonary Center Boston University School of Medicine Boston, MA United States; 6 Department of Learning Health Sciences University of Michigan School of Medicine Ann Arbor, MI United States; 7 Department of Internal Medicine University of Michigan Ann Arbor, MI United States

**Keywords:** shared decision-making, lung cancer, screening, clinical decision support, academic detailing, quality improvement, implementation

## Abstract

**Background:**

Lung cancer risk and life expectancy vary substantially across patients eligible for low-dose computed tomography lung cancer screening (LCS), which has important consequences for optimizing LCS decisions for different patients. To account for this heterogeneity during decision-making, web-based decision support tools are needed to enable quick calculations and streamline the process of obtaining individualized information that more accurately informs patient-clinician LCS discussions. We created DecisionPrecision, a clinician-facing web-based decision support tool, to help tailor the LCS discussion to a patient’s individualized lung cancer risk and estimated net benefit.

**Objective:**

The objective of our study is to test two strategies for implementing DecisionPrecision in primary care at eight Veterans Affairs medical centers: a quality improvement (QI) training approach and academic detailing (AD).

**Methods:**

Phase 1 comprised a multisite, cluster randomized trial comparing the effectiveness of standard implementation (adding a link to DecisionPrecision in the electronic health record vs standard implementation plus the Learn, Engage, Act, and Process [LEAP] QI training program). The primary outcome measure was the use of DecisionPrecision at each site before versus after LEAP QI training. The second phase of the study examined the potential effectiveness of AD as an implementation strategy for DecisionPrecision at all 8 medical centers. Outcomes were assessed by comparing absolute tool use before and after AD visits and conducting semistructured interviews with a subset of primary care physicians (PCPs) following the AD visits.

**Results:**

Phase 1 findings showed that sites that participated in the LEAP QI training program used DecisionPrecision significantly more often than the standard implementation sites (tool used 190.3, SD 174.8 times on average over 6 months at LEAP sites vs 3.5 SD 3.7 at standard sites; *P*<.001). However, this finding was confounded by the lack of screening coordinators at standard implementation sites. In phase 2, there was no difference in the 6-month tool use between before and after AD (95% CI −5.06 to 6.40; *P*=.82). Follow-up interviews with PCPs indicated that the AD strategy increased provider awareness and appreciation for the benefits of the tool. However, other priorities and limited time prevented PCPs from using them during routine clinical visits.

**Conclusions:**

The phase 1 findings did not provide conclusive evidence of the benefit of a QI training approach for implementing a decision support tool for LCS among PCPs. In addition, phase 2 findings showed that our *light-touch*, single-visit AD strategy did not increase tool use. To enable tool use by PCPs, prediction-based tools must be fully automated and integrated into electronic health records, thereby helping providers personalize LCS discussions among their many competing demands. PCPs also need more time to engage in shared decision-making discussions with their patients.

**Trial Registration:**

ClinicalTrials.gov NCT02765412; https://clinicaltrials.gov/ct2/show/NCT02765412

## Introduction

### Background

National lung cancer screening (LCS) guidelines have consistently recommended shared decision-making (SDM) to inform patients about the pros and cons of low-dose computed tomography (LDCT) screening and individualized LCS decisions [1,2]. The Centers for Medicare and Medicaid Services require documentation of SDM before initiating LDCT screening for its covered population, a policy that is unique among screening decisions in primary care [3]. Thus, understanding how to best implement and optimize SDM for LCS has been an urgent task for all health systems and clinicians offering LCS to their eligible patient population.

A key approach to SDM is to communicate accurate information about each person’s potential to benefit from screening, especially if it meaningfully differs from the average summary results reported in trials. This is particularly important for LCS. Prior work examining the range of absolute benefits across all individuals enrolled in the National Lung Screening Trial demonstrated that conveying average population information can dramatically overstate or understate lung cancer mortality reduction in thousands of individuals [4]. This is because both lung cancer risk and life expectancy varied substantially across eligible patients, such that the average mortality benefit identified in the National Lung Screening Trial was driven upward by those at the highest risk, whereas many patients experienced a benefit that was far below the average [5,6]. Using prediction-based approaches to estimate individualized net benefits can support the communication of much more accurate information across individuals in a heterogeneous group of screening eligible individuals [7]. Such approaches form an inherently more patient-centered basis for SDM [8].

### Objective

However, web-based decision tools that enable quick calculations and intuitive data presentations are needed to streamline the process of obtaining individualized information and more accurately inform patient–clinician LCS discussions in routine practice [9]. However, implementing clinical decision support tools in routine clinical practice has been difficult to achieve. Patient-facing tools have shown promise in improving patients’ understanding of the criteria and procedural requirements for lung screening [10]; however, discussing the details of individualized risks and benefits with patients—a critical aspect of SDM—can be challenging for providers. Numerous barriers, including infrastructure limitations and clinicians’ perceptions of SDM taking too much time, have led to a lack of uptake in the integration of decision support interventions into standard clinical practice [11,12]. Therefore, the objective of our study is to test two strategies for implementing a prediction-based SDM tool for LCS (DecisionPrecision) [13] in primary care at eight Veterans Affairs (VA) medical centers: (1) a quality improvement (QI) training approach and (2) academic detailing (AD).

## Methods

### Overview

Our implementation efforts and evaluation of each took place in 2 phases. In phase 1, we used QI training as a strategy for implementing DecisionPrecision as part of a hybrid type 3 implementation study design [14]. Specifically, we used a multisite, cluster-based randomization trial to compare the effectiveness of standard implementation versus the effectiveness of standard implementation plus the Learn, Engage, Act, and Process (LEAP) QI training program [15]. The standard implementation comprised integrating a link to the tool into the VA computerized patient record system (CPRS) and providing educational materials on the tool to a local LCS champion.

Although we observed a substantial number of tool uses, primarily by dedicated screening coordinators (as described in the following sections), results from phase 1 suggested that neither LEAP nor standard implementation contributed to the absolute number of tool uses at a site by primary care providers (PCPs). Consequently, phase 2 of the study switched to a different implementation strategy—namely, AD—and the study design transitioned to a hybrid type 2 implementation study, which comprised coprimary aims: (1) to determine the effectiveness of the clinical intervention (ie, DecisionPrecision) on key outcomes and (2) to determine the potential effectiveness of AD as an implementation strategy for the intervention. In phase 2, the focus of this study was on the second coprimary aim. Findings from our evaluation of the effectiveness of the tool *in the LCS decision* (first coprimary aim) will be described elsewhere.

The primary outcome measure for both phases is the use of DecisionPrecision at the site level (ie, the absolute number of tool uses at a site over a specific period). This was the best measure of the reach of our implementation strategies that was feasible, given the constraints we faced: use of a standalone tool that was deidentified and not connected to the health record and did not allow tracking of which clinicians and patients were associated with specific tool uses. This more ecologic site–level measure does not precisely fit the definition of reach in the reach, effectiveness, adoption, implementation, and maintenance (RE-AIM) framework [16], which defines reach as “the absolute number, proportion, and representativeness of individuals who are willing to participate in a given initiative, intervention, or program.” Moreover, although providers are the ones who must decide if they are willing to use the tool, it is the patients who we are ultimately trying to influence with tool use. Thus, we felt that it was more important to measure the number of tool uses rather than the number of providers using the tool. If 1 provider used the tool once and another provider used it 50 times, we were interested in the fact that the tool was used on 51 patients rather than the fact that it was used by 2 providers. A separate paper will focus on the effect of tool use on patient uptake of LCS (*effectiveness* from the RE-AIM framework).

In accordance with requirements of the journal, the CONSORT-EHEALTH (Consolidated Standards of Reporting Trials of Electronic and Mobile Health Applications and online Telehealth) checklist for reporting the study’s methods and findings was completed (Multimedia Appendix 1).

### Common Methodology Across Phase 1 and Phase 2

#### Setting and Site Selection

We recruited 8 VA sites that participated in the Evaluation of the VA LCS Clinical Demonstration Project (LDCT demo) [17]. These sites were chosen as (1) they had an LCS program currently in place, and (2) as part of the LDCT demo, they used a standard set of clinical reminders built by the VA’s National Center for Health Promotion and Disease Prevention to identify patients eligible for LCS. Clinical reminders are embedded within the electronic health record of the Veterans Health Affairs (CPRS) and can prompt staff to screen patients, review or assess risk factors, or offer interventions and screenings that may be due for an individual patient. The use of these standardized reminders facilitates data collection on LCS eligibility, discussions, and decisions. These advantages provided an ideal situation for testing different implementation strategies for a decision support intervention.

Of these 8 sites, 7 (87%) agreed to participate in our implementation initiative, and 1 (13%) declined because of a competing program. To replace the eighth site, we used VA administrative data to identify sites that used the LCS clinical reminder in CPRS as an indicator of an active LCS program. One of these sites agreed to participate in the study.

#### Participants

To implement DecisionPrecision, we worked with clinical leaders from each site’s LCS program and screening coordinators when available. These core site team members enlisted others at their sites to help with implementation, including primary care leadership; PCPs; and team members from pulmonology, radiology, and oncology departments. Information technology and data security staff members were also engaged.

#### Intervention: DecisionPrecision

To meet the need for facilitating SDM and providing individualized information on LCS, we created DecisionPrecision, a provider-facing web-based decision support tool [13]. Our goal was to facilitate individualized and patient-centered SDM within the confines of a busy clinic environment. In particular, the tool seeks to quickly help PCPs tailor the LCS discussion based on the patient’s risk factor profile, more strongly encouraging screening for those with higher predicted lung cancer risk and potentially larger health gains with LCS. To this end, the tool provides the following: (1) individualized quantitative risk assessment of screening trade-offs, aided by an externally validated and accurate lung cancer risk prediction model [18], along with recommendation categories that clarify when screening is and is not preference sensitive [7]; (2) patient-friendly language for the provider to use with the patient; (3) patient-facing graphics, selected based on their ability to help patients understand personalized risks and benefits in a randomized survey experiment [19]; and (4) quick and easy documentation of personalized SDM after using the tool.

The final version of the DecisionPrecision tool tested in this implementation study was the result of multiple rounds of iterative changes that incorporated lessons from an observational field study of patient-clinician conversations in the absence of a decision support tool and iterative feedback and usability testing on multiple tool prototypes from decision aid researchers, PCPs, pulmonologists, screening coordinators, and patients. After phase 2, the tool also underwent extensive updates based on experiences and observations throughout the implementation project and additional feedback from clinicians, screening coordinators, and leadership. Screenshots of DecisionPrecision can be found in Multimedia Appendix 2.

### Ethics Approval

This project was categorized as QI and was, therefore, not formally reviewed by the institutional review boards of participating sites [20].

## Phase 1: Evaluation of a QI Implementation Strategy

### Phase 1 Methods

In phase 1, the 8 participating sites were randomized to either standard (S1, S2, S3, and S4) or enhanced implementation (ie, the LEAP QI training program: E1, E2, E3, and E4), stratified by the rate of clinician completion of the initial LCS clinical reminder (high vs low completion for patients eligible for LCS between May 2015 and June 2015). A CONSORT (Consolidated Standards of Reporting Trials) diagram summarizing the randomization of the sites is included in Multimedia Appendix 3. The characteristics of the randomized sites are included in Multimedia Appendix 4. Both standard and enhanced implementation strategies are described in detail in the following sections.

#### Standard Implementation

The standard implementation involved the following: (1) adding a tool link to the VA’s electronic health record CPRS and (2) promoting the tool via emails, conference calls, and meetings.

##### DecisionPrecision Access Within the Electronic Health Record

A link to the decision tool was embedded within the clinical reminders for PCPs at all sites between August 29, 2017, and October 4, 2017. The language associated with the link and the specific location of the link were established based on conversations with the site team to best fit the mechanisms for the LCS at the site.

##### Promotion of DecisionPrecision

All sites were asked to notify relevant providers about DecisionPrecision. Site leads were given a draft email to providers that could be easily tailored with site-specific information. This email included a brief description of the key features of the tool and a link to a brief YouTube video that describes the tool’s development (eg, how the algorithm was designed) and how to use the website. Educational materials on the tool were provided, including a sample risk assessment from the decision tool, a 1-page attachment explaining how to routinely and quickly use DecisionPrecision to personalize discussions about LCS, and a screenshot of the link in the CPRS clinical reminder. The site teams were asked to promote decision aid through key local leaders and staff meetings.

#### Enhanced Implementation

Enhanced implementation included all the components available in the standard implementation as well as QI training using the LEAP program. LEAP is a 26-week QI training program to engage frontline clinical teams in using a hands-on learning approach (see Multimedia Appendix 5 for a brief description of the LEAP curriculum). The core components include a structured curriculum that focuses on QI methods, a coaching and learning community, and a web-based platform for sharing videos and other resources. Training goals include gaining confidence in applying QI methods to improve the quality of care within the demands of everyday clinical practice and completing a QI project using plan-do-study-act principles.

Of the 4 sites randomized to LEAP, 3 (75%) participated from February to July 2017 (E1, E2, and E4), and 1 (25%) declined (E3) because of time constraints for the site leads. The participating sites established an interdisciplinary LEAP team comprising 2 to 10 participants. The teams developed and executed a project charter to complete a plan-do-study-act cycle of change related to enhancing SDM for LCS using DecisionPrecision. The team members participating in LEAP were provided with early access to a link to DecisionPrecision in March 2017 so they could access the site as part of their improvement projects.

The improvement project at 1 site (E1) was the development of a process within 1 patient-aligned care team, whereby DecisionPrecision was used to identify eligible patients at the highest risk of lung cancer and then inform the SDM conversation for at least one veteran each week during the LEAP improvement program. The project for the other 2 sites (E2 and E4), which had more centralized screening programs and full-time screening coordinators, was for the screening coordinator to test the use of DecisionPrecision with all new consults for LCS.

#### Evaluation Methods

Phase 1 used a hybrid trial type 3 design [14]. The primary purpose of this design was to evaluate the effectiveness of the implementation strategy, and our primary question was, *Does enhanced implementation work better than standard implementation for facilitating the use of DecisionPrecision?* Thus, the primary outcome measure for evaluating the effectiveness of the enhanced implementation strategies was the use of DecisionPrecision at each participating site. The absolute tool use data by site were obtained from the DecisionPrecision website. In March 2018, a dropdown box was added asking the provider to indicate their site. To identify sites before March 2018, we used data on IP addresses collected by the website for each record entered and linked each IP address to a study site based on validation against the site data and IP addresses collected after March 2018. The analysis of these data included descriptive statistics of tool use by site. We also conducted brief interviews to assess the clinicians’ impressions of the implementation strategy.

### Phase 1 Results

As noted under the *Setting and Site Selection* section, sites were selected for this study based on their participation in the VA’s LCS Clinical Demonstration Project to ensure that all sites had similar LCS programs. However, shortly after the start of the study, we observed that the sites had made some changes to their screening programs. Specifically, some sites had stopped the collection of data required to calculate lung cancer risk (specifically, detailed smoking histories and key data used by DecisionPrecision), some sites stopped the routine use of clinical reminders, some sites no longer used a screening coordinator for conducting SDM, and some sites no longer had a screening coordinator for performing any LCS tasks. As each of these changes had the potential to affect the use of DecisionPrecision, these changes across sites are summarized in Table 1.

Consequently, our findings for this phase are presented by comparing the standard implementation sites to three different groups of facilities: (1) the original 4 facilities randomized to enhanced implementation (*intention to treat*), (2) the 3 facilities that participated in the enhanced implementation program (*as treated*), and (3) the 3 facilities that had a full-time screening coordinator engaged in SDM discussions with patients (*key resource* scenario).

**Table 1:** Summary of important changes occurring across the study sites after randomization.

Changes	Sites randomized to standard implementation					Sites randomized to enhanced implementation with LEAP^a^			
	S1	S2	S3	S4	E1		E2	E3^b^	E4
Lung cancer screening clinical reminders (for providers)?	Limited	Yes	Limited	Yes	Limited		Yes	Yes	Yes
Screening coordinator for conducting shared decision-making?	No	No	No	No	No		Yes	Yes	Yes
Pack year reminder?	Limited	Yes	Limited	No	Yes		Yes	Yes	Yes

^a^LEAP: Learn, Engage, Act, and Process.

^b^After randomization, this site decided not to participate in LEAP but continued to participate in the overall trial.

Table 2 shows the data on tool use for all sites in the 6 months following the time at which they all had access to DecisionPrecision but before phase 2 (AD) was initiated in April 2018. It also indicates the sites in each comparison group.

**Table 2:** Tool use each month by site (number of patients).

Site	Enhanced^a^	LEAP^b,c^	Screening coordinator^d^	October 2017, n	November 2017, n	December 2017, n	January 2018, n	February 2018, n	March 2018, n	Total, n	Per month, mean (SD)
E1	✓	✓		7	1	5	2	4	4	23	3.8 (2.1)
E2	✓	✓	✓	54	80	52	74	72	85	417	69.5 (13.6)
E4	✓	✓	✓	42	47	40	40	30	34	233	38.8 (6.0)
E3	✓		✓	1	7	2	33	23	22	88	14.7 (13.2)
S1				2	0	0	0	0	0	2	0.3 (N/A^e^)
S2				1	0	0	0	0	0	1	0.2 (N/A)
S3				0	0	0	2	0	0	2	0.3 (N/A)
S4				3	2	1	2	0	1	9	1.5 (0.8)

^a^Randomized to enhanced implementation.

^b^LEAP: Learn, Engage, Act, and Process.

^c^Participated in LEAP QI training program.

^d^Staffed by a lung cancer screening coordinator.

^e^N/A: not applicable.

Table 3 presents the 6-month mean number of tool uses between the standard implementation sites versus the 3 comparison groups. All 3 comparisons showed significantly less tool use for the standard implementation sites. However, the presence of a screening coordinator at 3 of the enhanced implementation sites and none of the standard implementation sites greatly confounded these comparisons.

**Table 3:** Mean number of tool uses over 6 months: 4 standard implementation sites as compared with 3 ways of grouping intervention sites.

Comparisons	Tool uses: 6 months, mean (SD)^a^		Median difference *P* value^b^	Median difference (95% CI)^b^
	A sites	B sites		
Standard implementation sites (A) versus the original 4 facilities randomized to enhanced implementation (*intention to treat*; B_1_)	3.5 (3.7)	190.3 (174.8)	.03	155.5 (14-416)
Standard implementation sites (A) versus the 3 facilities that participated in the enhanced implementation program (*as treated)*; B_2_)	3.5 (3.7)	224.3 (197.1)	.049	231.0 (14-416)
Standard implementation sites (A) versus the 3 facilities that had a full-time screening coordinator engaged in SDM^c^ discussions with patients (*key resource* scenario; B_3_)	3.5 (3.7)	246.0 (164.9)	.049	231.0 (79-416)

^a^Total tool uses across all the sites in the group divided by the number of sites in the group.

^b^On the basis of the Wilcoxon rank-sum test using the medians of the differences (Hodges-Lehmann estimator) between sites in the 2 groups.

^c^SDM: shared decision-making.

### Phase 1 Discussion

Our phase 1 findings showed that sites that participated in the LEAP QI training program used DecisionPrecision significantly more often than the standard implementation sites. However, there is some indication that participation in LEAP was not the primary contributing reason for these findings, as the implementation arms were imbalanced with regard to the presence of a screening coordinator. As evidence, a site in the enhanced implementation group that did not participate in LEAP (E3) but had a screening coordinator adopted and used the tool. In addition, a site in the enhanced implementation group that did not adopt the tool in routine use (E1) was also the site in the group that no longer used a screening coordinator. Although we could not determine tool use by provider type (PCP vs screening coordinator) from the website data, the vast majority of tool uses at sites E2, E3, and E4 was by screening coordinators based on tool use data collected manually by the coordinators, which showed numbers comparable with those obtained from the website.

Although participation in the LEAP may have contributed to the increased use of the tool, we conclude that the existence of a screening coordinator likely played a much larger role in tool use. Therefore, the question remains whether the screening coordinators who adopted the tool would have used it to a lesser extent if they had not participated in LEAP. Although the site with a coordinator who did not participate in LEAP showed the lowest tool use of the 3 sites with coordinators, other data (not shown) showing tool use as a percentage of all eligible patients indicated that E3 had a comparable rate of tool use with that of E2 and E4.

In addition, feedback from one of the screening coordinators suggested that QI training was not key to implementing the tool:

Well, it [LEAP] seemed to be more tied towards quality process improvement, so it was helpful for that. When it comes to the decision precision tool, I’m not really sure if I can concretely tie it to that.

QI collaboratives, including internet-based videoconferencing adaptations, are a common approach to helping health care teams implement new initiatives or improve existing programs [21]. However, evidence for their success is mixed, including weaknesses in the reporting of methods and potential publication bias. Nevertheless, findings from several studies have shed light on some factors that are correlated with successful implementation. A study of 11 collaboratives focusing on 11 different topics found that innovation attributes, organizational support, innovative team culture, and professionals’ commitment to change are instrumental to perceived effectiveness [22]. With specific regard to an innovation’s attributes, the study found that the newer working methods were perceived by professionals as having relative beneﬁt, being compatible with norms and values, not difﬁcult to learn and implement, and leading to observable results, the more the implementation process was perceived as successful. This finding is certainly consistent with feedback from screening coordinators, who all perceived DecisionPrecision as doing an excellent job in conveying important information on risks and benefits to patients. They found that the tool was relatively easy to use and incorporate into their workflow.

A systematic review of QI collaboratives concluded that collaboratives reporting success generally addressed relatively straightforward aspects of care and had a strong evidence base [21]. The implementation of DecisionPrecision could be considered straightforward in that it required only the addition of a link in the electronic medical record and did not require any significant changes in procedures or workflows. In addition, the scientific evidence underlying a prediction-based approach to LCS is relatively strong.

Furthermore, findings from a study on the effect of a learning collaborative on colorectal cancer screening rates in primary care practices are also consistent with ours [23]. Specifically, the teams had difficulty spreading the change beyond the clinicians who participated in the collaboration:

Other clinicians in a practice tended not to be aware of or engaged in the CRC (colorectal cancer) improvement efforts, and teams tended to communicate poorly with the rest of the practice regarding QI plans.

As a result—as occurred in our case—other providers, most notably PCPs, were not engaged and did not adopt the tool.

Another similarity between our study and the colorectal cancer screening study was that the clinicians who participated in the collaboratives (screening coordinators in our case) were very motivated to use the tool to improve the LCS process, which may not be the case among clinicians who did not participate (eg, PCPs).

Whether LEAP had a significant impact on the absolute number of DecisionPrecision uses by the screening coordinators, the collaborative approach did not have the intended effect of engaging the broader community of PCPs in using the tool. Feedback from participants in LEAP suggested the potential utility of an alternative strategy, namely one that focuses on one-on-one conversations with clinicians about the tool. Of the 26 sessions of the LEAP program, 1 (4%) was devoted to presenting and discussing the evidence behind the tool and how to use it. One screening coordinator noted the following:

I think the only helpful parts of it [LEAP], when it came to trying to implement the DecisionPrecision tool, was talking with the team...about what the stratified risks really mean...how you can come up with things like personalized harms and having that shared decision-making conversations where things are more preference-based–understanding that piece was extremely helpful and I can say that now, hindsight being 20/20 and having done a ton of shared decision-making in the last couple of years, I don’t think I could’ve done it as effectively if I didn’t have the knowledge that [was] shared with us during the LEAP program.

On the basis of the phase 1 findings, we switched to a different implementation strategy in phase 2—namely, AD.

AD was selected as an implementation strategy to convey information directly and one on one to PCPs about the evidence behind prediction-based screening and explain how to use DecisionPrecision. As noted on the National Resource for AD website [24], busy clinicians need an accurate source of current data on the effectiveness of current interventions. However, they have many competing demands for their time. Trying to assemble current evidence from a continuous influx of research is incredibly challenging to do on one’s own. AD combines a one-on-one outreach approach with the best available evidence. We hired and trained a master’s student in public health to meet clinicians to assess individual needs and then offer tailored, evidence-based advice for using DecisionPrecision as part of the LCS SDM process.

## Phase 2: Academic Detailing

### Phase 2 Methods

Our AD strategy focused on directly engaging PCPs, in addition to screening coordinators. We decided to use this strategy at all 8 participating sites rather than randomize sites to AD versus standard implementation in an effort to conduct a more extensive formative evaluation of the AD process, which, to the best of our knowledge, has not before been used to promote the use of a prediction-based SDM tool.

The goal of AD was to encourage providers, through the use of the DecisionPrecision tool, to adopt a prediction-based approach to tailoring how strongly screening is encouraged (based on estimated net benefit for the individual and consideration of how preference sensitive the decision is) to thereby facilitate a brief *everyday SDM* discussion and make decision-making more patient centered [7,8]. AD site visits were offered to all sites; of the 8 sites, 7 (87%) agreed to the site detailing visits. One of the sites underwent substantial workforce changes during the study and opted not to participate in the AD. Heads of primary care were asked to send emails to PCPs announcing the detailed visit and the purpose of the visit and encourage providers to participate in one-on-one detailing.

AD materials, which were developed and available for use during meetings, included a 4-page visual abstract of the evidence behind, benefits of, and key features of DecisionPrecision; a pocket card on how to use the tool; a CPRS clinical reminder screenshots and tool link handout; a handout on how to copy a templated description of the SDM discussion into the medical record; a list of references (in case of questions or concerns about the evidence); and a business card that included the URL to the DecisionPrecision website. The key information presented during these meetings were (1) how using a prediction-based approach for LCS can improve quality of care and (2) how to use the DecisionPrecision tool with eligible veterans to inform more patient-centered SDM and tailor screening encouragement during SDM discussions. At the end of each detailing session, the academic detailer asked for a provider’s commitment to using the decision tool in the next 1 to 2 weeks and for permission to follow up with them 3 to 4 weeks after the detailing visit [25]. See Multimedia Appendix 6 for a summary of the characteristics of the AD strategy per the published recommendations.

We conducted semistructured phone interviews with a sample of PCPs for 2 to 4 weeks following their AD visits. The interviews included questions on the utility of the AD visit, use of DecisionPrecision since the visit, usefulness of the tool, ease of tool use, challenges in using the tool, and suggestions for improving the tool. Audio-recorded interviews lasted approximately 20 minutes and were transcribed verbatim.

#### Evaluation Methods

As noted in the *Introduction* section, this paper presents data on the potential effectiveness of AD as a strategy for promoting the *use* of DecisionPrecision. Data on the effectiveness of the tool as a clinical intervention for improving the quality of LCS decisions have been presented elsewhere. The effectiveness of AD as an implementation strategy was assessed by (1) examining tool use following the AD visits and (2) conducting semistructured interviews with a subset of PCPs following their participation in AD visits.

##### Analysis of Tool Use

We conducted an interrupted time-series analysis to determine whether there was a difference in the overall tool use between the 6 months following the initiation of AD and the 6 months following the initiation of enhanced implementation (and before AD). We fitted a linear mixed model with the study period as the fixed effect of interest and a random intercept for each site.

##### Post-AD Interviews

We used NVivo (version 12; QSR International) to conduct an inductive thematic content analysis of the postdetailing interviews, searching for themes that emerged from the qualitative data. Team discussion of the findings led to agreement on the common themes, which included the major barriers to tool use and the features of the tool that the providers found to be beneficial.

### Phase 2 Results

We examined 105 PCPs at the 7 participating sites from June to October 2018 (E3 chose not to participate). Each site visit lasted 2 to 3 days, except for E1, where visits occurred over 2 months. The academic detailer met providers in primary care clinics, primary care resident clinics, and community-based outpatient clinics. Snowball sampling was used to identify providers before and during the site visit. Individual meetings were tailored to provider needs in terms of both content and duration. The duration of the meetings ranged from 4 to 40 minutes, with a mean duration of 13 minutes. Most meetings were one on one; however, a few meetings were with 2 to 3 providers simultaneously.

#### Tool Use by Site

Table 4 shows data on absolute tool use for the 6 months following the initiation of AD for all sites participating in AD. For comparison purposes, the last 2 columns of the table also show the total and average tool use for a similar period (6 months) before the AD intervention.

**Table 4:** Monthly tool use at seven sites participating in AD^a,b^ (number of patients).

Sites	Before AD							Total 6 months after AD		Per month after AD, mean (SD)		Total 6 months before AD^c^		Per month before AD,^c^ mean (SD)
	October 2018, n	November 2018, n	December 2018, n	January 2019, n	February 2019, n	March 2019, n								
All sites	84	90	114	137	137	153	715		119.2 (27.9)		687		114.5 (12.1)	
E1	1	4	2	2	4	1	14		2.3 (1.4)		23		3.8 (2.1)	
E2	52	62	65	72	73	94	418		69.7 (14.2)		417		69.5 (13.6)	
E4	20	20	45	51	51	37	224		37.3 (14.4)		233		38.8 (6.0)	
S1	4	4	1	5	2	7	23		3.8 (2.1)		2		0.3 (N/A^d^)	
S2	0	0	0	1	0	1	2		0.3 (N/A)		1		0.2 (N/A)	
S3	7	0	1	4	7	13	32		5.3 (4.4)		2		0.3 (N/A)	
S4	0	0	0	2	0	0	2		0.3 (N/A)		9		1.5 (0.8)	

^a^AD: academic detailing.

^b^The site designations, Enhanced Implementation and Standard Implementation, are not relevant for phase 2 as all sites received academic detailing; however, the labeling was maintained for linking to the phase 1 data (last 2 columns).

^c^Pre-AD months: October 2017 to March 2018; data are pulled from Table 2.

^d^N/A: not available.

An interrupted time-series analysis showed no significant difference in tool use between pre- and post-AD periods (95% CI 5.06-6.40, fewer tool uses after AD to more tool uses after AD; *P*=.82). Thus, it appears that the introduction of AD as an implementation strategy did not encourage substantial additional tool use (see Multimedia Appendix 7 for detailed results of this analysis).

#### Interview Responses

Of the 105 PCPs who participated in the AD, 83 (79%) provided their contact information for follow-up purposes. Of the 83 providers contacted, 33 (40%) participated in the post-AD follow-up interviews. Virtually unanimously, the participants felt that the AD visit helped provide them with an explanation of the tool’s purpose, the science underlying its development, and how to use it. Respondents appreciated the concise presentation and opportunity for a one-on-one discussion to walk them through the tool. Regarding the tool itself, many saw value in the tool’s ability to (1) shape clinician feelings about the LCS and (2) guide a useful approach to LCS discussions. They also felt that the tool enhanced their ability to share information about individualized risks and the pros and cons of screening. However, at follow-up, a few PCPs (6/33, 18%) used the tool with an actual patient. Limited time in the clinic was perceived as a key barrier by almost all the PCPs. Most PCPs reported needing 1 to 2 minutes to discuss LCS but frequently voiced not having even 1 to 2 minutes during a visit because of patient-specific needs that were a higher priority (eg, acute complaints) or organizational priorities (eg, performance measures). Similarly, having to input clinical data on risk factors into the tool was seen as a significant barrier to tool use as it added more time to the visit.

### Phase 2 Discussion

In phase 2, there was no difference in tool use between before and after AD. Follow-up interviews with PCPs indicated that the AD strategy increased provider awareness and appreciation for the benefits of the tool. However, other priorities and limited time prevented PCPs from using them during routine clinical visits.

We decided to pursue AD as a strategy because of the consistent literature documenting the effectiveness of this approach for aligning clinician behavior with evidence-based best practices [26-29]. Others have emphasized that for eHealth to optimize preventive care, electronic risk factor data need to be seen as relevant and useful by PCPs [30]. Before our AD intervention, we had limited success in engaging PCPs in considering the important role of overall lung cancer when making LCS decisions. We needed a strategy that could help us have meaningful conversations with frontline clinicians making daily decisions about LCS.

Although rooted in a strong evidence base, prediction-based approaches to decision-making about cancer screening are relatively novel and unfamiliar to many clinicians [31,32]. There are potential cognitive challenges with moving from SDM that conveys population average information to a one-size-fits-all approach for all patients who meet eligibility criteria and toward a prediction-based approach that tailors the strength of the recommendation based on the degree of estimated net benefit for each eligible individual. Given the frequency of cancer screening decisions in primary care and how entrenched practice styles and decision-making can be for such practices, it was unclear whether a brief AD intervention would be able to successfully convey the rationale for a prediction-based approach. Most AD studies have focused on medication prescribing, and few such studies used detailing to modify a decision-making approach to a commonly delivered category of service such as cancer screening [29].

On the basis of feedback from follow-up interviews with participating providers, AD allowed us to meaningfully engage dozens of PCPs across 7 sites. At the very least, these providers are now familiar with the prediction-based approach to SDM for LCS, the tool, and how it can be used in a busy primary care setting. Interview responses suggest that we have changed the way some clinicians think about decision-making for LCS, especially their understanding of the utility of a prediction-based approach to screening decisions. Nevertheless, our light-touch AD did not result in the routine use of the tool among PCPs in our study sample, primarily because of time constraints. As others have noted, the incentives (and disincentives) in our health care system will need to change if providers are to have sufficient time to engage in SDM [33].

## General Discussion

### Limitations

The limitations of this study include the small sample size (8 sites). Other studies have emphasized the importance of examining whether specific components of multicomponent implementation strategies have stronger associations with absolute tool use than other components in an effort to streamline these strategies to be more time and cost-effective [34]. A larger sample size would have enabled us to examine whether specific components of LEAP were associated with the use of DecisionPrecision. An additional limitation was that enhanced implementation was confounded by the presence of a screening coordinator, making it impossible to attribute the findings to the enhanced implementation strategy alone. In phase 2, the AD intervention was based on a single site visit and a single one-on-one conversation with PCPs rather than multiple reinforcing visits or a longitudinal relationship with PCPs.

### Conclusions

The phase 1 findings do not provide conclusive evidence of the benefit of a QI training approach for implementing a decision support tool for LCS among PCPs. Screening coordinators in the study used the tool frequently, and it is possible that the LEAP program helped them adopt the tool. However, other factors may have contributed to tool use, including the coordinators’ perception of the added benefit of using the tool as part of their responsibility for educating patients on LCS and the relative ease of incorporating tool use into their workflow.

As PCPs were not engaged in the phase 1 implementation strategy, the phase 2 implementation strategy—AD—targeted these clinicians. On the basis of our experience with phase 1, the focus of the AD approach was to educate PCPs on the benefits of tool use and discuss the best ways of incorporating it into their clinical practice. However, even when PCPs see value in a prediction-based approach to LCS decision-making and a tool to support that approach, they face major challenges in implementing it in a busy primary care clinic. This was a consistent finding across all study sites. Thus, in terms of the RE-AIM framework, we feel that the adoption (willingness to initiate a program) and reach (willingness to use) of our standalone DecisionPrecision tool, if left unchanged, is likely to be limited among PCPs within other health care settings as well.

One implication of these findings for implementing decision support tools for LCS—and potentially other cancer screening tools—is that QI as an implementation strategy may not be helpful; instead, the focus of implementation should be on working with individual clinicians and screening coordinators to promote tool use. Screening coordinators bought into the rationale for using the tool *and* were able to adopt and use it routinely (high awareness and good reach among coordinators). However, our *light-touch*, single-visit AD strategy did not affect tool use among PCPs, although feedback from PCPs suggested that this strategy did achieve our goal of increasing provider awareness and appreciation of the benefits of the tool (awareness but limited adoption and reach among PCPs). Other priorities and limited time prevented PCPs from using them during routine primary care clinic visits. These barriers point to the second implication of our findings; namely, prediction-based SDM tools need to be automated as much as possible for use in primary care to better integrate into workflows and help PCPs more quickly understand how to prioritize LCS discussions among other competing demands [35]. Regarding the latter need, an ongoing Agency for Health Research and Quality–funded project is addressing this barrier by automating predictions and integrating the DecisionPrecision tool within multiple electronic health records, including Epic, Cerner, and CPRS health record systems. CPRS is still used in most VA health systems at present before a planned transition to Cerner [36-39].

## Appendix

Multimedia Appendix 1 CONSORT-EHEALTH checklist (V 1.6.1).

Multimedia Appendix 2 DecisionPrecision screenshots.

Multimedia Appendix 3 CONSORT (Consolidated Standards of Reporting Trials) diagram, phase 1.

Multimedia Appendix 4 Site characteristics.

Multimedia Appendix 5 Learn, Engage, Act, and Process (LEAP) curriculum.

Multimedia Appendix 6 Characteristics of the academic detailing strategy.

Multimedia Appendix 7 Results of the interrupted time-series analysis.

## Abbreviations

AD: academic detailing
CONSORT: Consolidated Standards of Reporting Trials
CONSORT-EHEALTH: Consolidated Standards of Reporting Trials of Electronic and Mobile Health Applications and online Telehealth
CPRS: computerized patient record system
LCS: lung cancer screening
LDCT: low-dose computed tomography
LEAP: Learn, Engage, Act, and Process
PCP: primary care physician
QI: quality improvement
RE-AIM: reach, effectiveness, adoption, implementation, and maintenance
SDM: shared decision-making
VA: Veterans Affairs
