# Supplementary material for: Implementation of a Web-Based Tool for Shared Decision-making in Lung Cancer Screening: Mixed Methods Quality Improvement Evaluation
Source: JMIR Hum Factors. 2022 Apr 1;9(2):e32399. doi: 10.2196/32399 (PMC9015752; doi:10.2196/32399)
Supplement: Multimedia Appendix 2 [file humanfactors_v9i2e32399_app2.docx]

**Multimedia Appendix 2: DecisionPrecision Screenshots**


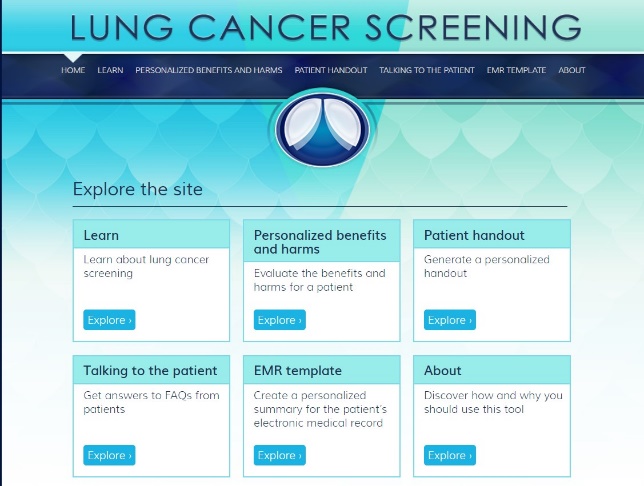
Home screen –

Example Personalization output –


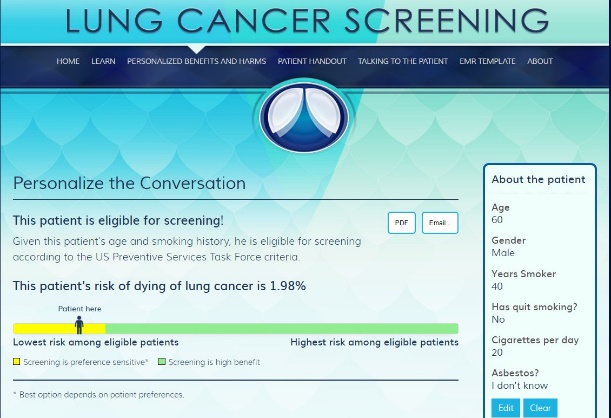


Example Risk-benefit Chart -


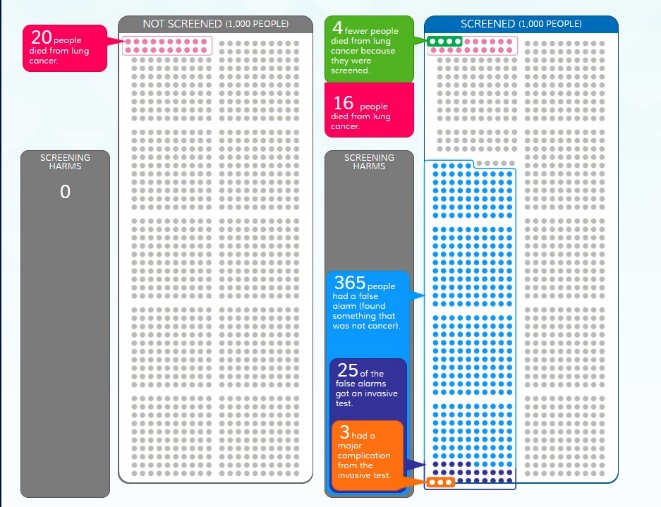


An updated version of this prediction-based SDM tool for LCS can be found at [www.screenlc.com](http://www.screenlc.com)
