# Supplementary material for: Implementation of a Web-Based Tool for Shared Decision-making in Lung Cancer Screening: Mixed Methods Quality Improvement Evaluation
Source: JMIR Hum Factors. 2022 Apr 1;9(2):e32399. doi: 10.2196/32399 (PMC9015752; doi:10.2196/32399)
Supplement: Multimedia Appendix 3 [file humanfactors_v9i2e32399_app3.doc]

**Multimedia Appendix 3.**

**Allocation**

**Analysis**

**Follow-Up**

**Enrollment**

Assessed for eligibility (n= 9)

Excluded (n= 1)

♦  Declined to participate (n= 1)

Analyzed (n= 4)

Lost to follow-up (give reasons) (n= 0)

Allocated to standard implementation (n= 4)

♦Received standard implementation (n= 4)

Lost to follow-up (give reasons) (n= 0)

Allocated to enhanced implementation (n= 4)

♦Received allocated intervention (n= 3)

♦Did not receive allocated intervention (due to time constraints) (n= 1)

Analyzed (n= 3, 4)
(Separate analyses were conducted with and without the site that did not participate in enhanced implementation)

Randomized (n= 8)

**CONSORT Flow Diagram for Site Randomization, Phase 1**
