# Supplementary material for: Implementation of a Web-Based Tool for Shared Decision-making in Lung Cancer Screening: Mixed Methods Quality Improvement Evaluation
Source: JMIR Hum Factors. 2022 Apr 1;9(2):e32399. doi: 10.2196/32399 (PMC9015752; doi:10.2196/32399)
Supplement: Multimedia Appendix 4 [file humanfactors_v9i2e32399_app4.docx]

|  | **S1** | **S2** | **S3** | **S4** | **E1** | **E2** | **E3** | **E4** |
| --- | --- | --- | --- | --- | --- | --- | --- | --- |
| **Geographic Location** | Midwest | Midwest | Northeast | Northeast | Midwest | Southeast | Southeast | Northwest |
| **Age of patients (%)** |  |  |  |  |  |  |  |  |
| 55-64 | 19.91 | 15.26 | 18.88 | 14.09 | 17.42 | 20.42 | 22.29 | 18.04 |
| 65-74 | 32.88 | 37.17 | 25.48 | 31.76 | 37.87 | 27.79 | 31.62 | 35.22 |
| 75-84 | 11.11 | 15.27 | 11.18 | 16.39 | 11.03 | 9.65 | 9.80 | 12.14 |
| **Race/Ethnicity** |  |  |  |  |  |  |  |  |
| % White | 76 | 82 | 48 | 84 | 75 | 52 | 54 | 75 |
| % Hispanic | 1 | 1 | 15 | 2 | 2 | 2 | 2 | 3 |
| **Gender (% Male)** | 91 | 93 | 88 | 93 | 91 | 87 | 88 | 91 |
| **Workforce (FTEE^a^)** |  |  |  |  |  |  |  |  |
| Hematology/ Oncology | 1.72 | 3.77 | 4.04 | 1.21 | 3.20 | 1.31 | 3.32 | 3.25 |
| PCPs | 34.6 | 52.7 | 36.7 | 28.8 | 38.9 | 53.0 | 55.6 | 55.8 |
| Pulmonology /Critical Care | 3.96 | 2.45 | 5.59 | 4.30 | 4.72 | 2.91 | 2.73 | 4.89 |
| Radiology | 6.14 | 17.25 | 12.95 | 3.38 | 13.44 | 10.97 | 14.62 | 13.02 |
| Thoracic Surgery | 0.54 | 7.51 | 2.36 | 0.12 | 2.22 | 0.68 | 2.27 | 2.72 |
| **Complexity^b^** | 1B | 1A | 1A | 1C | 1B | 1A | 1A | 1A |
| **No. of outpatient visits per year** | 583,495 | 931,383 | 735,380 | 474,449 | 630,107 | 934,308 | 785,216 | 948,323 |
| **Avg no. of LDCT quarterly** | 0.92 | 41.25 | 2.5 | 0.00 | 0.83 | 26.5 | 17.42 | 40.92 |
| **PCPs detailed** | 16 | 20 | 17 | 11 | 10 | 18 | 0 | 33 |
| **Completion rate of LCS clinical reminder during LCS demonstration project**  **(2012-2016)** | 62.8 | 13.8 | 79.1 | 49.1 | 20.3 | 28.4 | 54.0 | 68.6 |

**Multimedia Appendix 4: Site Characteristics (2017)**

^a^FTEE = Full time employee equivalent

^b^The Facility Complexity Model classifies VHA facilities at levels 1a, 1b, 1c, 2, or 3 with level 1a being the most complex and level 3 being the least complex. These levels are determined by a multitude of factors including but not limited to ICU Level and Operative Complexity Level, Number of residents, Research dollars, and Complex Clinical Programs.
