# Supplementary figures and images for: Implementation of a Web-Based Tool for Shared Decision-making in Lung Cancer Screening: Mixed Methods Quality Improvement Evaluation
Source: JMIR Hum Factors. 2022 Apr 1;9(2):e32399. doi: 10.2196/32399 (PMC9015752; doi:10.2196/32399)

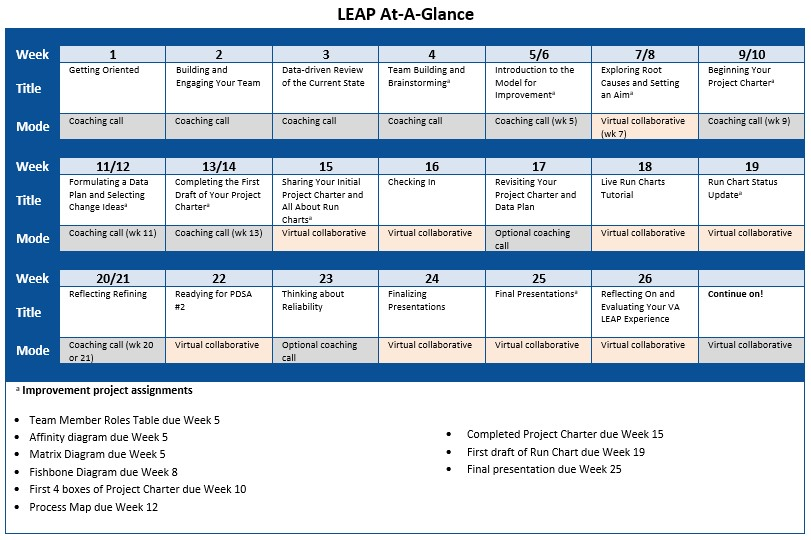

Supplement: Multimedia Appendix 5 [file humanfactors_v9i2e32399_app5.png]
