# Supplementary material for: Implementation of a Web-Based Tool for Shared Decision-making in Lung Cancer Screening: Mixed Methods Quality Improvement Evaluation
Source: JMIR Hum Factors. 2022 Apr 1;9(2):e32399. doi: 10.2196/32399 (PMC9015752; doi:10.2196/32399)
Supplement: Multimedia Appendix 6 [file humanfactors_v9i2e32399_app6.docx]

***Multimedia Appendix 6: Characteristics of Academic Detailing Strategy***

| **Characteristic** | **Description** |
| --- | --- |
| **Content of Visits** |  |
| - Clinician education | Explanation of the evidence for and benefits of a prediction-based approach to SDM for LCS. |
| - Recommendations about a practice change | Recommendation of how to use the tool with eligible patients to estimate net benefit for the individual based on their risk factors, determine how preference sensitive the decision is, and tailor how strongly screening is encouraged during the SDM discussion based on this individualized information; tool demonstration |
| - Tailoring | Identifying and addressing any specific needs or concerns. |
| **Nature of In-person visits** |  |
| - Number of visits/providers | 1 |
| - Duration of each visit | 4-40 minutes (mean = 13 minutes) |
| - Audience | Primary care providers |
| **Type of contact other than in-person** |  |
| - E-mail | One follow-up e-mail requesting a phone conversation with interview |
| - Phone | One follow-up phone conversation |
| **Qualifications/background** | Master’s in public health student. Additional training included: |
|  | - Intensive self-education on risk-based lung cancer screening, tool development, and tool use; mock detailing encounters |
|  | - Two-day training session (National Resource Center for Academic Detailing) |
| **Primary Outcome** | Number of tool uses at participating sites in the six-months following on-site visits compared to tool uses during same six months in prior year |
