# Supplementary material for: Implementation of a Web-Based Tool for Shared Decision-making in Lung Cancer Screening: Mixed Methods Quality Improvement Evaluation
Source: JMIR Hum Factors. 2022 Apr 1;9(2):e32399. doi: 10.2196/32399 (PMC9015752; doi:10.2196/32399)
Supplement: Multimedia Appendix 7 [file humanfactors_v9i2e32399_app7.docx]

**Multimedia Appendix 7: Tool Use Interrupted Time Series Analysis**

This analysis includes a single fixed effect for the two six-month time periods of interest and a random effect for site. Period A is the reference category and is from October 2017-March 2018; Period B is from October 2018-March 2019; and Period X is anytime outside Periods A or B.

|  | **n** | | |
| --- | --- | --- | --- |
| *Predictors* | *Estimates* | *CI* | *p* |
| (Intercept) | 16.36 | -0.85 – 33.56 | 0.062 |
| six_month_period (B) | **0.67** | **-5.06 – 6.40** | 0.820 |
| six_month_period (X) | -0.48 | -5.07 – 4.11 | 0.838 |
| **Random Effects** | | | |
| σ^2^ | 179.45 | | |
| τ_00_ _sta3n_ | 509.52 | | |
| ICC | 0.74 | | |
| N _sta3n_ | 7 | | |
| Observations | 231 | | |
| Marginal R^2^ / Conditional R^2^ | 0.000 / 0.740 | | |
|  |  | | |

Across all sites, there was an average of 0.67 more tool uses each month at each site from October 2018-March 2019 compared to October 2017-March 2018, with a 95% confidence interval of -5.06 to 6.40 tool, indicating no evidence of a difference between these 6-month periods.
